# Risk factors for vascular catheter-related bloodstream infections in pediatric intensive care units

**DOI:** 10.5935/0103-507X.20180066

**Published:** 2018

**Authors:** Fabiola Peixoto Ferreira La Torre, Gabriel Baldanzi, Eduardo Juan Troster

**Affiliations:** 1 Departamento de Pediatria, Faculdade de Ciências Médicas, Santa Casa de São Paulo - São Paulo (SP), Brasil.; 2 Unidade de Terapia Intensiva Pediátrica, A. C. Camargo Cancer Center - São Paulo (SP), Brasil.; 3 Instituto de Tratamento de Câncer Infantil, Hospital das Clínicas, Faculdade de Medicina, Universidade de São Paulo - São Paulo (SP), Brasil.; 4 Escola de Medicina, Faculdade Israelita de Ciências da Saúde Albert Einstein - São Paulo (SP), Brasil.

**Keywords:** Catheter-related infections, Catheterization, central venous/adverse effects, Cross infection, Risk factors, Intensive care units, pediatric

## Abstract

**Objectives:**

To determine the risk factors for acquiring central line-associated blood stream infections (CLABSI) in pediatric intensive care units and to investigate the incidence and etiology of CLABSI in pediatric intensive care units with different profiles.

**Methods:**

The study was a prospective cohort study in three hospitals. One of the hospitals is a large metropolitan public hospital with two pediatric intensive care units and a total of nineteen pediatric intensive care unit beds, another is a regional hospital with eight pediatric intensive care unit beds, and the third is a private hospital with fifteen beds. Patients between the ages of 1 month old and 18 years old who used a central venous catheter for over 24 hours were included. We recorded patients’ daily progress. General patient and catheter-related data were collected and used as variables. All the data were analyzed using Statistical Package for Social Science (SPSS), version 13.0, to compare patients with CLABSI with or without risk factors.

**Results:**

A total of 728 patients were admitted to the pediatric intensive care units, and 170 had a central line in place for at least 24 hours. The median age was 32 months, and 97 (57%) of the patients were males. The CLABSI incidence rate was 3.9/1000 central venous catheter-days. The incidence among hospitals varied from 1.6 to 6.6. The overall mortality rate was 11.1%, and the CLABSI and non-CLABSI mortality rates were 12.9% and 10.7%, respectively. In multivariate analysis, independent risk factors for CLABSI were a longer duration of central venous catheter use (OR: 1.07; 95%CI 1.00 - 1.14; p = 0.019) and the use of more than one central venous catheter at once (OR: 2.59; 95%CI 1.17 - 5.73; p = 0.048).

**Conclusion:**

A longer duration of central venous catheter use and the use of more than one central venous catheter at once were the main risk factors for CLABSI in pediatric intensive care units.

## INTRODUCTION

Even though central venous catheter (CVC) are indispensable in pediatric intensive care units (PICU), central line-associated blood stream infections (CLABSI) are associated with an increase in attributed mortality from 4% to 37%, in addition to prolonged hospital stays and increased hospital costs.^(1,2)^

Central line-associated blood stream infections rates differ between developed and developing countries, which have rates of 1.2 and 6.5 cases/1000 CVC, respectively.^(3-7)^ In the adult population, factors associated with CLABSIs include the site of CVC insertion and the duration of CVC use, the presence or absence of underlying disease, the severity score, the presence or absence of immunosuppression, the type of dressing used and whether an aseptic insertion technique is used.^(8-11)^ However, information on factors associated with CLABSI in the pediatric population is limited, especially in developing countries.

Factors associated with CLABSI in children are probably different than those in adults, considering that the reasons for PICU admission are distinct, that CVC for pediatric patients have multiples sizes, and that obtaining venous access in infants is often challenging.

The objective of this study was to identify CLABSI risk factors in the PICU population and to investigate the incidence and etiology of CLABSI in PICU with different profiles.

## METHODS

This multicenter prospective surveillance study took place in three hospitals in São Paulo, Brazil. The *Santa Casa de Sao Paulo* is a tertiary public teaching hospital with two PICUs: a 13-bed level III unit with clinical and surgical patients, including cardiac surgery and renal and liver transplant patients, and a 6-bed level II unit with clinical patients and patients recovering from minor surgery. The *Hospital Municipal Prof. Dr. Alípio Correa Neto* is a secondary hospital with an 8-bed level II unit, and the *Hospital Israelita Albert Einstein* is a private service with a 15-bed level III unit with clinical and surgical patients, including cardiac surgery patients. The study was approved by the Ethics Committee at all three hospitals, and the requirement for written informed consent was waived.

All patients admitted to any of the four PICUs from the age of 1 month to the age of 18 years who had a central line access for a period of over 24 hours, were included in the study from August 2012 to July 2013. Patients with tunneled or implanted venous catheters were not included.

Data were prospectively collected daily using a standardized format by the principal investigator or by trained hospital staff for all patients admitted to the PICU meeting the inclusion criteria. Patients were followed until discharge to another hospital or until two days after transfer to a ward in the same hospital. The total number of patient admissions in each unit was also monitored during the study period.

For each patient, the records included demographic data and data on chronic conditions; the length of the PICU and hospital stay; mortality; the use of mechanical ventilation; vasoactive drug administration; hemodynamic instability; requirements for other indwelling devices; previous infections; exposure to antibiotics, antifungals or blood products; metabolic disorders; the number of CVC lumens and site (e.g., the jugular vein); and the CVC insertion procedure (e.g., the professional who performed it).

The current Centers for Disease Control and Prevention/National Healthcare Safety Network criteria for CLABSI were used.^(12)^ A CLABSI was defined as a clinically and/or laboratory-confirmed bloodstream infection in a patient with a central line or umbilical catheter placed for at least 48 hours with no infection diagnosed at another site. Bloodstream infections diagnosed 48 hours after CVC removal were also considered CLABSI. Any central access device that terminated at or close to the heart or great vessel was considered a CVC.

### Statistical analysis

Categorical variables are presented as proportions and were compared using the Chi-squared test or Fisher’s exact test when the expected subgroup size was smaller than five. Continuous variables are presented as medians and ranges and were analyzed with the Mann-Whitney test. The number of needle insertions was categorized as one or more than one insertion. Variables with a p value <.05 in univariate analysis were included in multivariate logistic regression analysis. Odds ratios were calculated with a 95% confidence interval. The data were analyzed using Statistical Package for the Social Sciences (SPSS), version 13.0 (Statistical Package for the Social Sciences, IBM, NY, USA).

The main indicator calculated was the occurrence of laboratory CLABSI. It was calculated as follows:

$$\begin{array}{c} \mathrm{Primary}\;\mathrm{laboratory}\;\mathrm{blood}\;\mathrm{current}\;\inf\mathrm{ection}\;=\;\mathrm{number}\;\mathrm{of}\\ \mathrm{new}\;\mathrm{cases}\;\mathrm{of}\;\mathrm{primary}\;\mathrm{laboratory}\;\mathrm{blood}\;\mathrm{current}\;\inf\mathrm{ection}/\\ \mathrm{number}\;\mathrm{of}\;\mathrm{patients}\;\mathrm{with}\;\mathrm{CVC}\times 1000 \end{array}$$

The clinical CLABSI indicator was also calculated, and its formula is:

$$\begin{array}{c} \mathrm{Primary}\;\mathrm{clinical}\;\mathrm{blood}\;\mathrm{current}\;\inf\mathrm{ection}\;=\;\mathrm{number}\;\mathrm{of}\\ \mathrm{new}\;\mathrm{cases}\;\mathrm{of}\;\mathrm{primary}\;\mathrm{clinical}\;\mathrm{blood}\;\mathrm{current}\;\inf\mathrm{ection}/\\ \mathrm{number}\;\mathrm{of}\;\mathrm{patients}\;\mathrm{with}\;\mathrm{CVC}\;\times 1000 \end{array}$$

One variable that can be used to aid in the interpretation of infection indicators is the rate of use of CVC. This variable indicates the degree to which the sample analyzed is exposed to the risk of infection.

Therefore, the incidence of vascular catheter infection is the total number of infections of CVC divided by the total number of exits multiplied by 100. The density of CLABSI was calculated by the following formula:

$$\begin{array}{c} \mathrm{Number}\;\mathrm{of}\;\mathrm{patients}\;\mathrm{with}\;\mathrm{CLABSI}/\mathrm{CVC}\;\\ \mathrm{per}\;\mathrm{day}\times 1000 \end{array}$$

## RESULTS

During the study period, 728 patients were admitted to the four PICUs, and 170 had a central line in place for 24 hours. The median age was 32 months, 97 (57%) of the patients were males and 116 (68%) of the patients had an underlying medical condition (e.g., congenital cardiopathy). The patient demographic data are detailed in table 1. CLABSI were diagnosed in 31 cases, representing 18% of the patients with CVCs, with an overall incidence rate of 3.9/1000 CVC-days. The incidence among hospitals varied from 1.6 to 6.6 (Table 2). The overall mortality rate was 11.1%, and the CLABSI and non-CLABSI mortality rates were 12.9% and 10.7%, respectively (p = 0.48).

**Table 1:** Demographic characteristics

	CLABSI group 31 patients	Non-CLABSI group 139 patients	p value
Male	22 (71)	75 (54)	0.08
Age (months)	27 (1 - 158)	30 (1 - 192)	0.334
Deaths	4 (13)	15 (11)	0.73
PIM II	6.35	5.4	0.133
Length of PICU stay (days)	13 (±11)	11.5 (±8)	0.172
Chronic disease	23 (19.8)	93 (80.2)	0.287

CLABSI - central line-associated blood stream infection; PIM II - Pediatric Index of Mortality Score II; PICU - pediatric intensive care unit. The values are expressed as n (%), mean (min - max), mean, or mean (standard deviation).

**Table 2:** Central line-associated blood stream infection per study center

	CLABSI numbern (%)	CLABSI incidence rate/1000 CVC-days
Level III PICU SCSP	3 (9.6)	1.6
Level II PICU SCSP	21 (67.7)	6.6
HACN	6 (19.3)	4.9
HIAE	1 (3.2)	2.4
Total	31 (100)	3.9

CLABSI - central line-associated blood stream infection; CVC - central venous catheter; PICU - pediatric intensive care unit; SCSP - Santa Casa de São Paulo; HACN - *Hospital Alípio Correa Neto*; HIAE - *Hospital Israelita Albert Einstein*.

Among the 31 CLABSI, 28 were laboratory-confirmed with a positive blood culture. Gram-negative bacteria were isolated in 72% of cases (*Klebsiella spp.* 21%; *Acinetobacter spp.* 18%), while gram-positive bacteria and *Candida spp*. were each isolated in 14% of cases.

Table 3 summarizes the main exposure variables studied. In univariate analysis, the variables associated with CLABSI were the presence of another indwelling device (e.g., a urinary catheter), infection diagnosed at PICU admission, exposure to blood products during the PICU stay, the use of antifungal drugs, potassium disturbance in the PICU, CVC insertion with more than a single needle insertion attempt, the use of more than one CVC at once, and a longer duration of CVC use. In multivariate analysis, the independent risk factors for CLABSI were a longer duration of CVC use (OR: 1.07; 95%CI 1.00 - 1.14; p = 0.019) and the use of more than one CVC at once (OR: 2.59; 95%CI1.17 - 5.73; p = 0.048) (Table 4).

**Table 3:** Main exposure variables studied

Exposure variable	CLABSI(31)	Non-CLABSI(139)	p value
PICU readmission	3 (9.7)	7 (5)	0.266
Underlying medical condition	23 (74.2)	93 (66.9)	0.28
Presence of another indwelling device	19 (61.3)	52 (37.4)	0.013*
Hemodialysis	1 (3.2)	9 (6.5)	0.425
Another infection site at PICU admission	27 (87.1)	48 (34.5)	0.012*
Blood product exposure	28 (90.3)	85 (61.2)	0.001*
Previous antibiotic exposure	31 (100)	127 (91.4)	0.82
Antifungal drugs	21 (67.7)	19 (13.7)	< 0.0001*
Sepsis (at admission and/or during PICU stay)	18 (58.1)	62 (44.6)	0.123
Hemodynamic instability	19 (61.3)	62 (44.6)	0.361
Vasoactive drug	23 (74.2)	97 (69.8)	0.401
Potassium disturbance	17 (54.8)	41 (29.5)	0.011*
Continuous sedation	28 (90.3)	127 (91.4)	0.540
Femoral catheter	10 (22)	29 (21)	0.172
More than one needle insertion	26 (84)	79 (58)	0.007
Fellow-inserted CVC	13 (41.9)	64 (46)	0.416
Ultrasound guidance	6(19.4)	29 (21)	0.528
Education on CVC maintenance	11(35.5)	54 (38.8)	0.447
More than one CVC at once	23 (74)	41 (29)	< 0.0001*
Duration of CVC use, median; [min-max] (days)	24 [2 - 111]	7 [1 - 45]	< 0.0001*

CLABSI - central line-associated blood stream infection; PICU - pediatric intensive care unit; CVC - central venous catheter;

* p < 0.05. The results are expressed as n (%) or median [min - max].

**Table 4:** Independent risk factors for central line-associated blood stream infection in multivariate analysis

	Adjusted odds ratio (95%CI)	p value
More than one CVC at once	2.59 (1.17 - 5.73)	0.019
Longer duration of CVC use	1.07 (1.00 - 1.14)	0.048

95%CI - 95% confidence interval; CVC - central venous catheter.

It is noted that infection density was highest in the university hospital, specifically in the central ICU, in this study; among the ICUs examined, this ICU is where most renal, hepatic and cardiac surgeries are performed, where there is greatest circulation of medical and multiprofessional specialties, and where the conditions of the patients are most severe.

## DISCUSSION

The results from this prospective surveillance study in four heterogeneous PICU reflect the CLABSI incidence rate and risk factors in a major city in South America. The incidence of 3.9/1000 CVC-days found in this study is higher than the rates in North America (1.2 - 2.15);^(3,4,7)^ rather, it is closer to European rates (1.7 - 3.7)^(5,13)^ and is lower than the rates reported in developing countries according to the last International Nosocomial Infection Consortium.^(6)^ In Sao Paulo, the health care assistance is considered to be the most advanced in Brazil, but improvements are needed to reach the healthcare-associated infection rates of the United States and Canada.

Many possible risk factors were included in data collection, but only a longer duration of CVC use and the use of more than one CVC at once were statistically significantly associated with CLABSI in logistic regression analysis. A single-center case-control study in Brazil from Vilela et al. found the same risk factors in multivariate analysis but also found a protective effect of concomitant antibiotic use.^(14)^ A large study by Costello et al. that analyzed 3319 admissions to the pediatric cardiac intensive care unit found that a central venous line in place for ≥ 7 days and exposure to ≥ 3 blood products units were associated with CLABSI (OR: 6.06 and 5.56, respectively).^(15)^ In our study, blood product exposure was associated with CLABSI in univariate analysis but not in logistic regression. However, the present study considered patients to be exposed to blood products regardless of the number of units received. Therefore, 61.2% of patients without CLABSI and 90.3% with CLABSI were considered to have been exposed to blood in our study compared to the 13.9% and 100%, respectively, in the Costello study.^(15)^ Elward and Fraser reported an increased risk of CLABSIs in patients who had received a high number of packed red blood cell transfusions (adjusted OR: 1.2; 95%CI 1.1 - 1.4; p < 0.001).^(16)^ Despite these findings, a meta-analysis including 17 trials did not find a reduced risk of healthcare-associated infections overall with a restrictive transfusion strategy.^(17)^

Adherence to barrier precautions during catheter insertion is desirable to reduce the chance of infection.^(18)^ All CVC were placed using maximal sterile barrier precautions, including cap, mask, sterile gown, sterile gloves and sterile full body drape. Despite the precautions taken, having more than CVC at once was an independent risk factor for CLABSI in multivariate analysis, and the presence of another indwelling device was associated with CLABSI in univariate analysis. Elward and Fraser found that a higher number of arterial catheter-days was associated with nosocomial bloodstream infection with an adjusted OR of 5.7 (95%CI 3.4 - 9.8).^(16)^ Odetola et al. reported a relative risk of 5.5 (95%CI 1.3 - 23.0) with two intravascular access devices and 12.4 (95%CI 3.0 - 50.1) with three or more devices.^(19)^ Other devices, such as Foleys catheters and chest tubes, were also associated with an increased risk of CLABSI.^(15)^ The association between the number of indwelling devices and infection is biologically plausible, as every skin and mucosal disruption is a potential infection source. Therefore, any unnecessary device must be removed as soon as possible.

To assure a low incidence of infections, close adherence to a bundle of care is warranted. An insertion and maintenance bundle of care for CVC together with rigorous reporting reduced CLABSI rates by 71% in neonatal intensive care units in North Carolina.^(20)^ Moreover, systematic surveillance of CLABSI is essential to reduce infection rates.^(18,21)^

The most common pathogens isolated were gram-negative bacteria (72%), which is consistent with the results of previous studies.^(5,22)^ Due to the risk of multiple antibiotic resistance, these microorganisms are of increasing concern. Among all the gram-negative isolates, 25% were resistant to carbapenem. This result could impact antibiotic choice for septic shock patients with suspected CLABSI until the organism antibiogram is available.

The PICUs included were chosen because of their convenience to the investigators who worked in the hospitals; therefore, the results may not reflect all the units in the Sao Paulo area. In addition, CLABSI incidence rates varied considerably across hospitals, showing the heterogeneity of the PICUs. On the other hand, the consistency of the results of this study with results reported in the literature suggests that a longer duration of CVC use and the use of more than one CVC at once are likely important risk factors for CLABSIs in most, if not all, PICUs. One strength of this study is that data were collected daily and prospectively over a year, making the results reliable.

One of the prevention strategies for CLABSI is the education of professionals responsible for catheter insertion and maintenance; the use of maximum precaution barriers during insertion and preparation of the skin with 0.5% chlorhexidine are also important strategies. Pediatric data on CLABSI risk factors are scarce, especially in developing countries, where the healthcare professional-to-patient ratio is lower than that in developed countries and the education levels are irregular. In our study, only 38% of the nursing staff received continuing education on CVC maintenance.

No statistical association was found between CLABSI occurrence and the practitioner who inserted the catheter or between CLABSI occurrence and the handling of catheters by professionals who had or had not undergone continuing education to prevent CLABSI. Continuing education was considered some kind of theoretical and practical training related to the prevention of CVC-related bloodstream infections.^(23)^

This finding was surprising, since in the literature, continuing education programs have already been evaluated as factors to improve the prevention of infection. Perhaps the small number of people in training caused a bias in the outcome.

In many PICUs, assessing the need for all catheters is part of the daily routine. However, the final decision to remove the lines mostly depends on the individual and the clinic.

Daily evaluation of the need for central catheter maintenance and discussion of this need during multidisciplinary visits is important to enable the removal of catheters that are no longer needed.^(23)^ In our study, the duration of CVC use was a risk factor for CLABSI in PICU. When CVC use was prolonged by one day, the chance of the patient presenting with a CLABSI increased 7-fold according to the multivariate analysis. Future studies should evaluate systematic interventions to shorten the duration of CVC use and reduce the need for multiple venous access devices.

These results may stimulate the implementation of CLABSI prevention strategies such as the creation of a Catheter Group to standardize routines for the insertion, maintenance and withdrawal of CVC. In addition, these results may provide guidance on the careful use of catheters and the importance of adherence to and assistance with standardized protocols for care with catheters. Another important strategy suggested by this study is the incorporation of knowledge into the practice of hand washing, which will reduce the prevalence of bloodstream infections as well as infections in general.

It is considered important to carry out specific studies with different types of ICUs, since there are variations in the length of stay and, consequently, the duration of catheter use among ICU types, which in turn cause variations in infection rates related to invasive procedures.

Consideration should be given to cases involving variables that present a relative risk for CLABSI with statistical significance. Such cases may be candidates for pharmacological intervention for CLABSI prevention, such as the use of catheters impregnated with antibiotics, the use of dressings impregnated with antiseptics, and catheter blockade with antibiotics or ethanol.

## CONCLUSION

The present study made possible a deeper analysis of pediatric central line-associated blood stream infections in São Paulo local pediatric intensive care units, which have incidence rates greater than those in North America. A longer duration of central venous catheter use and the use of more than one central venous catheter at once were the main risk factors for CLABSI in the pediatric intensive care units in this study. Bundles of care must focus on these factors to be more effective.
